# Perpetuating Inequality: Junior Women Do Not See Queen Bee Behavior as Negative but Are Nonetheless Negatively Affected by It

**DOI:** 10.3389/fpsyg.2018.01690

**Published:** 2018-09-20

**Authors:** Naomi Sterk, Loes Meeussen, Colette Van Laar

**Affiliations:** ^1^Center for Social and Cultural Psychology, KU Leuven, Leuven, Belgium; ^2^Postdoctoral Fellowship, Research Foundation–Flanders, Brussels, Belgium

**Keywords:** self-group distancing, sexism, queen bee effects, negative affect, ambiguity, bias

## Abstract

Previous research has revealed that women may attempt to avoid negative gender stereotypes in organizations through self-group distancing, or “queen bee”, behaviors: emphasizing masculine qualities, distancing themselves from other women, and legitimizing organizational inequality. Factors that increase self-group distancing have been identified (e.g., existing discrimination and low group identification), but it is unknown how self-group distancing by an ingroup leader is perceived by and affects subordinates of the negatively stereotyped group. In the current study, female participants received ambiguous negative feedback from a male versus female leader displaying queen bee-type versus neutral behavior. As expected, a male leader displaying queen bee-type behavior was seen as having less positive intent than a male leader displaying neutral behavior, which in turn increased how sexist he was perceived to be. A female leader displaying queen bee (vs. neutral) behavior was not seen as having less positive intent, which thus did not indirectly influence perceived sexism. Behavior of both male and female leaders did affect junior women: participants exposed to a leader displaying queen bee-type behavior reported more anger, sadness, and anxiety than participants exposed to a leader displaying neutral behavior. These data provide further evidence that simply adding more women or minorities in key senior positions is insufficient to change inequality if bias in the organization is not tackled. Specifically, exposure to gender inequality can steer female leaders to endorse–rather than change–stereotypes about women, and this behavior is particularly consequential because it (a) might not be recognized as bias and (b) exerts negative effects.

## Introduction

Despite significant changes in social equality policies and legislation, women remain underrepresented in various fields and in higher positions in society. Within the largest companies in the European Union, women comprise only 5% of CEOs and 23% of board members (European Commission, 2016). These numbers are comparable to those in the United States, in which women in the largest companies comprise 5% of CEOs and 21% of board members (Catalyst, 2017). In Europe as well as in the United States, however, these numbers do mark a slight increase in the proportion of women as CEOs and in boards. For example, in 2012, 14% of board members in the largest EU companies and 17% of board members in the largest United States companies were women (Catalyst, 2012; European Commission, 2012). It is often believed that such an increase in female leadership will undoubtedly lead to increased gender equality, as women climbing the organizational ladder are presumed to lower organizational bias, actively resist structural inequalities for example in selection procedures, mentor other women (Stout et al., 2011), and create a more identity safe organizational climate for other women (Purdie-Vaughns et al., 2008). Moreover, the mere presence of women in leadership roles should–by providing real life exemplars of female leaders–initiate change in how the leadership role is perceived (Eagly and Karau, 2002; Koenig and Eagly, 2014). Also, in theory, an increasing number of women in leadership positions should attenuate the traditional ‘masculine’ stereotype of leadership that exists (Sczesny, 2003).

Drawing on research on the Queen Bee (QB) phenomenon, we maintain, however, that it is not certain that other women will automatically benefit from female leaders in top positions. In the current paper we aim to show that when female leaders display QB behavior, this in reality *negatively* affects other (junior) women. Such negative effects occur because QB behavior can look similar to sexism and exert similar effects, but might be less likely to be recognized as sexism due to the source being female, hereby impairing effective regulation by the receiver. We here outline these ideas.

Women who advance into higher positions often experience barriers due to their gender. As the figures above show, they often find themselves as one of only a few women in (the top of) male-dominated organizations. Female leaders also often have to walk a tightrope between meeting the demands placed on them due to their leader role (e.g., evidencing agentic qualities) and meeting the demands placed on them due to their gender role (e.g., evidencing high communal qualities) (Eagly and Karau, 2002; Eagly and Sczesny, 2009). Moreover, women advancing into higher positions still face bias and gender stereotypes, which can induce social identity threat. Some women navigate this by engaging in self-group distancing: a process whereby members of stigmatized groups cope with inequality by disengaging with the stigmatized group and assimilating into the non-stigmatized group. Groups for which this has been found include women, the elderly, ethnic minorities, and sexual minorities (Eguchi, 2009; Derks et al., 2011a,b, 2015; Weiss and Lang, 2012; see Derks et al., 2016 for a discussion). Self-group distancing can thus be seen as an individual strategy to resolve social identity threat and to restore a devalued identity (Branscombe et al., 1999a). Female self-group distancing has been coined “Queen Bee” (QB) behavior (Staines et al., 1974), and is characterized by a masculine self-presentation, legitimizing the status quo (e.g., denying gender inequality in the workplace and opposing measures aimed at reducing gender inequality), and underlining dissimilarities to other women (Derks et al., 2016).

Although in recent years more insight has been gathered into factors linked to the development of QB behavior (experiencing gender inequality, low gender identification), it is unknown how QB behavior is interpreted by and affects those who are exposed to it. QB behavior can be seen as ambiguously negative behavior, showing similarities to modern forms of sexism. Unlike traditional forms of sexism, which are more overt and hostile, modern sexism is more subtle and ambiguous. Like QB behavior, modern sexism is characterized by a denial of continued gender discrimination and opposition to women’s demands, such as a lack of support for measures aimed at reducing gender equality (Swim et al., 1995; Tougas et al., 1995). Moreover, QB behavior can extend beyond a passive rejection of the ingroup (i.e., absence of acceptance) to actively devaluing ingroup members (e.g., claiming women have lower career commitment than men; Ellemers et al., 2004; Kaiser and Spalding, 2015). Thus, while QB behavior and sexism are distinct in their underlying concerns and motivations (with QB behavior being a self-regulation strategy, driven by identity devaluation concerns that are a *response* to social inequality such as sexism), QB behavior and modern sexism do show strong outward similarities. However, no research has experimentally examined these similarities and related these similarities to outcomes for those exposed to QB behavior. We thus set out to examine how junior women perceive and are affected by QB behavior. Specifically, we expected to show: (1) that QB behavior is less likely to be perceived as bias because the source of this behavior is a member of the negatively stereotyped group, (2) that despite not being seen as intentionally negative or as sexist, QB behavior negatively affects women confronted by it, and (3) that the ambiguity accompanying perception of this behavior impairs regulatory strategies.

### How Do Women Perceive Male and Female Leaders Displaying QB(-Type) Behavior?

The first aim of this research is to show that QB behavior is less likely to be perceived as bias due to the source of this behavior being female. Although QB behavior appears similar to modern forms of sexism, it is unlikely that QB behavior will be perceived as being driven by negative or sexist intentions. As QB behavior describes a phenomenon occurring in women, for a female subordinate the source of this behavior will be an ingroup member. Ingroup leaders are generally viewed in a more positive light than outgroup leaders, even if they express a preference for members of the outgroup (Duck and Fielding, 2003). This ingroup-outgroup difference can be explained by a phenomenon known as the intergroup sensitivity effect: the tendency for people to be less skeptical of criticism or the source of this criticism if it is coming from an ingroup member than from an outgroup member (Hornsey et al., 2002). This lower skepticism follows from people tending to ascribe positive intent to someone expressing themselves negatively about their ingroup: They believe that the speaker intends to be constructive and means well (Hornsey and Imani, 2004). Furthermore, an ingroup member would be a non-prototypical source of bias, and bias from a non-prototypical source (e.g., sexism from a female, racism from an ethnic minority) is less likely to be recognized as such (Baron et al., 1991; Inman and Baron, 1996; Inman et al., 1998; Cunningham et al., 2009). Because of this non-prototypicality, behavior that would otherwise be perceived as negative might not necessarily be seen as such when the source of this behavior is an ingroup member. We thus expect that perceptions of a leader displaying QB behavior are influenced by the fact that the source of this behavior is female, and expect the same kind of behavior to be perceived differently if the source of this behavior is male. [When we talk about QB-type behavior from a male leader, we are talking about the same behaviors but enacted by a male leader. Hence by definition this behavior cannot be labeled as queen bee behavior, as queen bee behavior describes self-group distancing in women and can therefore only be displayed by women–a man cannot distance himself from the female ingroup because this is not his ingroup. Therefore, for comprehension purposes, we refer to QB behavior enacted by a male leader as ‘QB-type’ behavior. When referring to a male and female leader simultaneously we use the term ‘QB(-type)’ behavior.]. Our first hypothesis is thus that a male leader displaying QB-type (vs. neutral) behavior will be perceived as having less positive intent toward women and will therefore be seen as sexist, whereas this will not be the case for a female leader. Put differently, QB behavior by a female leader will go less recognized as a possible instance of sexism because the female source will be seen as having positive intent toward women.

### How Are Women Affected by QB(-Type) Behavior?

The second aim of this research is to demonstrate that despite not being seen as bias, QB behavior is likely to negatively affect women confronted by it. The “why” of what is being said might be perceived as positive (constructive), but the “what” of what is being said is still similar to modern gender bias. Exposure to bias in its traditional, more blatant, sense has negative consequences such as psychological distress (Klonoff et al., 2000; Szymanski et al., 2009) and negative affect (Wang et al., 2012). Bias does not have to be traditional or obvious, however, to exert negative effects (Barreto and Ellemers, 2005; Salvatore and Shelton, 2007; Murphy et al., 2013). For example, Barreto and Ellemers (2005) exposed participants to statements reflecting either modern sexism (e.g., “discrimination against women is no longer a problem”) or traditional blatant sexism (e.g., “women are generally not as smart as men”). The results showed that although participants were not as likely to perceive modern (vs. traditional) sexist statements as sexist, they did show increased anxiety compared to the traditional sexism condition. Our second hypothesis, therefore, is that participants who are exposed to QB(-type) behavior (vs. neutral behavior) will suffer negative consequences (measured through increased negative affect), both when the source of this behavior is male and when the source of this behavior is female (Hypothesis 2).

The third and last aim of this research is to demonstrate that the regulation of negative affect following exposure to QB(-type) behavior will be impaired when the source of this behavior is an ingroup member (female) rather than an outgroup member (male). One way people can regulate negative affect is through identification with the stigmatized group. The rejection-identification model (Branscombe et al., 1999b) posits that although attributions of experiences to bias negatively affect well-being through feelings of rejection, such attributions can also protect well-being through identification with the stigmatized group. In other words, group identification can attenuate feelings of rejection caused by bias because one can still feel included in the stigmatized group. Group identification can also be protective because high group identifiers are more attentive to bias and more likely to recognize bias when it occurs, making it more possible to regulate its negative effects (Operario and Fiske, 2001; Major et al., 2003). As we argue below, both of these mechanisms through which group identification can protect well-being in the face of possible bias are likely to be impaired in the context of QB behavior. Firstly, QB behavior involves rejection stemming from a fellow member of the stigmatized group, so protective effects of gender identification with the stigmatized group are less likely to occur. Secondly, we argue that QB behavior is ambiguous regarding attributions to bias because the source of this behavior is an ingroup member. When bias is ambiguous or unclear, higher identification with the stigmatized group does not protect against negative effects (Major et al., 2003; Dardenne et al., 2007). When the source of QB-type behavior is male and thus an outgroup member, both of these protective functions of group identification are presumably not impaired. Accordingly, our third hypothesis is that women who are more highly identified with their gender group will be protected from negative effects of QB(-type) behavior, but only when the source of this behavior is male. Put differently, we expect that exposure to QB(-type) behavior will not increase negative affect in women who are highly identified with their gender group when the source of this behavior is male–while this will be the case when the source is female (Hypothesis 3).

In sum, we predict that QB behavior is less likely to be perceived as bias because the source of this behavior is female, that despite not being seen as intentionally negative or as sexist QB behavior negatively affects women confronted by it, and that the ingroup source of this behavior–a female–impairs effective regulation of these negative effects.

## Materials and Methods

### Participants

Participants were 1st-year female Psychology students in Belgium, who participated in a study about ‘their perspective on the university and the future’ for course credits. Three of the 171 participants who completed the study were excluded from analyses: One participant was excluded for answering with only the most extreme values on each scale and two participants were excluded because they themselves indicated not having participated seriously^1^. The final sample consisted of 168 participants with a mean age of 18.4 years old (*SD* = 1.17, range 17–29). Most participants (92.3%) self-identified as Belgian and 17.4% (also) identified with another group (such as Dutch, Turkish, Moroccan). We performed *post hoc* power analyses using G^∗^Power (Faul et al., 2007) for each of our effects to test whether our sample provided sufficient power. An overview of all power estimates can be found in **Table 1**. Power was sufficient for all effects of interest unless specified in the results and discussion sections.

**Table 1:** Estimates for power obtained per effect.

	Test statistics	Power
**H1: Perception^a^**		

*Positive intent*		
Gender of leader (GL)	*F*(1,166) = 15.04, *p* < 0.001, $\eta_{p}^{2}$ = 0.08	97.1%
QB(-type) behavior (QB)	*F*(1,166) = 21.09, *p* < 0.001, $\eta_{p}^{2}$ = 0.11	99.5%
QB × GL	*F*(1,164) = 8.76, *p* = 0.004, $\eta_{p}^{2}$ = 0.05	83.7%
*Sexism*		
Gender of leader (GL)	*F*(1,166) = 19.51, *p* < 0.001, $\eta_{p}^{2}$ = 0.11	99.2%
QB(-type) behavior (QB)	*F*(1,166) = 16.90, *p* < 0.001, $\eta_{p}^{2}$ = 0.09	98.3%
QB × GL	*F*(1,164) = 2.36, *p* = 0.126, $\eta_{p}^{2}$ = 0.01	33.3%
**H2: Effects**		

*Anger*		
QB(-type) behavior	*F*(1,166) = 20.92, *p* < 0.001, $\eta_{p}^{2}$ = 0.11	99.5%
*Sadness*		
QB(-type) behavior	*F*(1,166) = 18.19, *p* < 0.001, $\eta_{p}^{2}$ = 0.10	98.9%
*Anxiety*		
QB(-type) behavior	*F*(1,166) = 5.20, *p* = 0.024, $\eta_{p}^{2}$ = 0.03	62%
**H3: Regulation**		

*Anger*		
QB × GL × gender identification	*F*(1,160) = 1.82, *p* = 0.179, $\eta_{p}^{2}$ = 0.01	25.4%
*Sadness*		
QB × GL × gender identification	*F*(1,160) = 1.40, *p* = 0.239, $\eta_{p}^{2}$= 0.01	25.4%
*Anxiety*		
QB × GL × gender identification	*F*(1,160) = 3.85, *p* = 0.052, $\eta_{p}^{2}$ = 0.02	45.3%


*^*a*^The current study achieved at least 80% power for the conditional indirect effect of QB-type behavior on perceived sexism in the male leader condition, as demonstrated by more than sufficient sample size (Fritz and MacKinnon, 2007)*.

### Design and Procedure

The study had a 2 (leader gender: male/female) × 2 (leader behavior: QB[-type]/control) between-participants design. Data were collected online during collective testing sessions in computer rooms at the university. The study was approved by the Ethics Committee of the University. Following informed consent, a baseline measure of gender identification (moderator variable) was taken at the start of the collective testing session, ostensibly as part of a first (unrelated) study. After this unrelated study, which assessed students’ attitudes toward the university and which took about 15 min, participants were asked to imagine they had been working at a company for a short time (type of business not specified) and were presented with the manipulation of leader behavior (QB[-type] vs. control). Subsequently, participants answered the manipulation checks and answered dependent measures and control variables: perceived sexism and perceived intent of the leader, negative affect, and demographics. The study took approximately 25 min.

### Leader Gender and Leader Behavior Manipulation

The manipulation of the gender of the leader and the manipulation of the leader behavior (QB[-type] behavior vs. control) was situated within a (contrived) company magazine presented to participants, which included an introduction from the CEO and the manipulations in the form of a column. The purpose of this introduction was to provide participants with implicit information about the organizational context, namely a male-dominated organization (photo of male-only board of directors, statement that the company “is now 324 man strong”)–the context in which QB behavior is most likely to arise and in which junior women are most likely to be exposed to QB behavior (Derks et al., 2011a,b). Following this foreword, participants read a column designed to manipulate leader gender and leader behavior (see **Supplementary Materials** for full manipulation). The column was ostensibly written by their leader Luc or Marie (leader gender manipulation), in which their leader discussed the organization and his/her motivation for working there. As outlined below, QB(-type) behavior (vs. control) was manipulated by incorporating the following three general indicators of the QB phenomenon (Derks et al., 2016): (1) masculine self-description, (2) endorsement of gender stereotypes, and (3) denial of gender discrimination in the organization. All three were included together as they together have been defined as QB behavior and because we wanted to create a manipulation that was strong enough for a student sample (as they are as yet less attuned to the workplace) imagining a situation reading a single vignette.

Firstly, in all conditions the leader claimed that three characteristics were important for achieving success and emphasized that he/she had these characteristics. These characteristics were selected on the basis of a pretest to be similar in positive valence, but to be either highly *masculine* or *neutral*: highly masculine in the QB-(type) conditions (willingness to take risks, focus on results, and being strong) and neutral in the control conditions (being responsible, flexible, and sincere). Secondly, in the QB(-type) behavior conditions the leader (subtly) *endorsed gender stereotypes* by linking a masculine work environment with a no-nonsense work environment, the implication being that a feminine work environment is *not* a no-nonsense work environment. Thirdly, the leader in the QB-type behavior conditions *denied gender discrimination*, implying that individual merit (competence) and not structural disadvantage is the reason there are hardly any women in the organization.

The combination of these three indicators read as follows in the QB(-type) behavior conditions: *“I sometimes get asked: ‘Doesn’t it bother you, almost only male employees?’ Why would that bother me? Because of the masculine work environment? I stayed here because I like a no nonsense work environment. Because it might be unfair? What’s unfair about selecting employees based on competence?”*

In the control conditions these sentences read: *“I sometimes get asked: ‘Doesn’t it bother you, working for one company for such a long time?’ Why would that bother me? Because the work environment stays the same? I stayed here because I like this work environment. Because it’s hard work? What dream doesn’t require hard work?”*

After reading this company magazine, all participants received ambiguous negative feedback from their leader. The content of this feedback was identical across conditions and was added in order to make the situation more self-relevant for participants. The participant was told that the position in which her manager had started his or her career at the company was opening up soon, that this higher position was a good fit for the participant, and that the participant had expressed her interest in this position to her manager a few days ago. Participants were then shown the following ambiguous response from their leader: *“Thank you for your email and your interest in the function as assistant project leader, it is indeed a nice position. I have to tell you though that I’m not sure you will be accepted. So check if you want to put your time into that, or maybe think about it some more.”*

### Measures

Unless otherwise indicated, items were answered on a 7-point scale from (1) strongly disagree to (7) strongly agree. Measures are scored such that higher scores indicate stronger scores on the concept.

#### Perceived Positive Intent of the Leader

Two items measured perceived positive intent of the leader: “Luc/Marie has my best interests at heart” and “Luc/Marie has women’s best interests at heart” (*r* = 0.62, *p* < 0.001). The correct name (Luc for a male leader and Marie for a female leader) was inserted by the Lime Survey program depending on whether the participant’s leader was male or female.

#### Perceived Sexism of the Leader

To measure perceived sexism of the leader, participants were asked to indicate the extent to which they agreed with two items (correct name inserted by the Lime Survey program): “Luc/Marie is sexist” and “Luc/Marie acts belittling toward women” (*r* = 0.43, *p* < 0.001).

#### Negative Affect

We assessed three types of negative affect using items adapted from the PANAS scales (Watson et al., 1988). Participants were asked to imagine how they would feel in the presented situation, using a 7-point Likert scale from (1) not at all to (7) very much. Three items measured anger (angry, annoyed, and hostile, α = 0.87), four items measured sadness (down, sad, dissatisfied, and unhappy, α = 0.88) and four items measured anxiety (anxious, tense, nervous, and afraid, α = 0.85).

#### Gender Identification

To examine how gender identification altered responses we assessed gender identification using the ‘identity centrality’ subscale of the hierarchical model of ingroup identification (Leach et al., 2008). This subscale consists of the following three items: “Being a woman is an important part of how I see myself”; “The fact that I am a woman is an important part of my identity”; and “I often think about the fact that I am a woman” (α = 0.76). Gender identification was assessed at the beginning of the collective session as part of an ostensible separate study.

Means and standard deviations of all measures per condition as well as cohen’s *d* are provided in **Table 2**.^2^

**Table 2:** Descriptive statistics by leader gender and leader behavior.

		Control	QB(-type)	Cohen’s *d*
Perceived positive intent	Male leader	3.84 (1.06)	2.61 (1.05)	1.17
	Female leader	3.97 (1.13)	3.76 (1.19)	0.18
	Cohen’s *d*	0.18	1.04	
Perceived sexism	Male leader	3.19 (1.16)	4.20 (1.55)	0.73
	Female leader	2.68 (0.94)	3.09 (1.29)	0.37
	Cohen’s *d*	0.49	0.77	
Anger	Male leader	2.90 (1.40)	3.77 (1.39)	0.62
	Female leader	2.29 (1.02)	3.08 (1.22)	0.71
	Cohen’s *d*	0.51	0.52	
Sadness	Male leader	2.91 (1.07)	3.52 (1.11)	0.56
	Female leader	2.42 (1.05)	3.08 (0.88)	0.67
	Cohen’s *d*	0.46	0.43	
Anxiety	Male leader	3.60 (1.30)	3.84 (1.16)	0.20
	Female leader	3.20 (1.04)	3.73 (1.19)	0.48
	Cohen’s *d*	0.15	0.18	
Gender identification	Male leader	5.03 (1.15)	5.31 (0.89)	
	Female leader	5.19 (1.03)	5.31 (1.11)	


*Statistics presented are mean scores; standard deviations are presented between brackets. Cohen’s d is given for each comparison in the cell below (means for the male and female leader within each level of the behavior leader condition) or in the cell to the right-hand side (means for the control and QB[-type] behavior conditions within each level of the gender leader condition) of the corresponding means*.

## Results

### Initial Checks

Initial checks showed that the variance for perceived sexism of the leader was not equal across the conditions. An adjusted rank transformation test (ART) was performed in order to see if this heterogeneity of variance for perceived sexism affected the results. The ART is a non-parametric test suitable for analyzing interactions (Leys and Schumann, 2010). Data are adjusted and rank transformed, after which the adjusted data are analyzed with factorial ANOVA. The results obtained using ART did not differ from the results obtained using ANOVA, and thus for ease of interpretation we report the results obtained using ANOVA. The statistics for the ART analyses are available in the **Supplementary Materials**. There were no differences between conditions on gender identification, *F*(3,164) = 0.69, *p* = 0.561, demonstrating that randomization was successful.

### How Do Participants Perceive Male and Female Leaders Displaying QB(-Type) Behavior?

#### Perceived Positive Intent

We first examined to what extent participants saw their leader as having positive intent toward women. Consistent with expectations, participants in the QB(-type) conditions saw their leader as having less positive intent (*M* = 3.08, *SD* = 1.24) than did participants in the control conditions (*M* = 3.91, *SD* = 1.09), *F*(1,166) = 21.09, *p* < 0.001, $\eta_{p}^{2}$ = 0.11. The interaction effect between leader behavior and leader gender was also significant, *F*(1,164) = 8.76, *p* = 0.004, $\eta_{p}^{2}$ = 0.05. As expected, the male leader in the QB-type condition was perceived as having less positive intent (*M* = 2.61, *SD* = 1.05) than the male leader in the control condition (*M* = 3.84, *SD* = 1.06), *F*(1,164) = 26.87, *p* < 0.001, $\eta_{p}^{2}$ = 0.14. The female leader was perceived as having equally positive intent whether she evidenced QB behavior or not, *F*(1,164) = 0.72, *p* = 0.399, $\eta_{p}^{2}$ = 0.004.

#### Perceived Sexism: Direct Effects

Next we examined to what extent participants saw their leader as sexist. In general, participants saw the male leader as more sexist (*M* = 3.74, *SD* = 1.47) than the female leader (*M* = 2.85, *SD* = 1.11), *F*(1,166) = 19.51, *p* < 0.001, $\eta_{p}^{2}$ = 0.11. There was also a main effect of QB(-type) behavior on perceived sexism: participants in the QB(-type) conditions saw their leader as more sexist (*M* = 3.74, *SD* = 1.54) than did participants in the control conditions (*M* = 2.91, *SD* = 1.07), *F*(1,166) = 16.90, *p* < 0.001, $\eta_{p}^{2}$ = 0.09. Contrary to expectations, the overall interaction effect between leader behavior and leader gender was not significant, *F*(1,164) = 2.36, *p* = 0.126, $\eta_{p}^{2}$ = 0.01. An examination of the predicted slopes showed that the predicted simple main effect of QB-type behavior was significant in the male leader condition, *F*(1,164) = 14.19, *p* < 0.001, $\eta_{p}^{2}$ = 0.08, with the male leader being seen as more sexist in the QB-type condition (*M* = 4.20, *SD* = 1.55) than in the control condition (*M* = 3.19, *SD* = 1.16). The simple main effect of QB behavior was not significant in the female leader condition, *F*(1,164) = 2.14, *p* = 0.146, $\eta_{p}^{2}$ = 0.01 (respective means *M* = 3.09, *SD* = 1.29 and *M* = 2.68, *SD* = 0.94). An alternative breakdown of this interaction showed that the simple main effect of leader gender was marginally significant in the control condition, *F*(1,164) = 3.63, *p* = 0.059, $\eta_{p}^{2}$ = 0.02, and significant in the QB(-type) condition, *F*(1,164) = 15.27, *p* < 0.001, $\eta_{p}^{2}$ = 0.09. Yet, the lack of a significant overall interaction and low power for this interaction (0.33) shows that these differences were not strong enough to conclude that differences in attributions to sexism between the QB(-type) and control condition depended on leader gender.

#### Perceived Sexism: Indirect Effects

**FIGURE 1:**
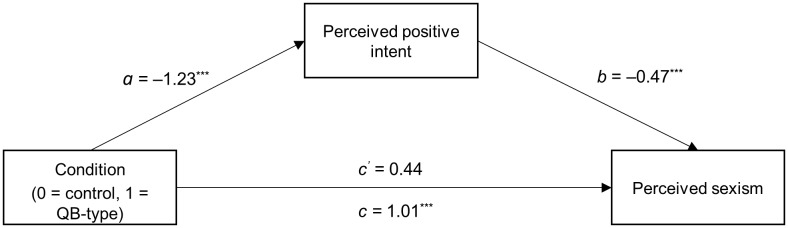
Mediation model for male leader showing effects of QB-type behavior on increased perceived sexism through reduced perceptions of positive intent (partially standardized regression coefficients, ^∗∗∗^*p* < 0.001).

Moderated-mediation analyses using the PROCESS macro for SPSS (Hayes, 2018, model 7) did, however, support the prediction that the lower perceptions of positive intent explained increased perceptions of sexism for the male leader displaying QB-type behavior: perceived sexism was entered as the dependent variable, leader behavior was entered as the predictor variable, perceived positive intent as the proposed mediator, and leader gender was added as the proposed moderator for the a′ – b′ relationship. The results of these analyses are summarized in **Table 3**. As expected, the moderated mediation was significant (index = -0.48, *SE* = 0.18, 95% CI [-0.84, -0.15]). Perceived positive intent fully mediated the effect of QB(-type) behavior on perceived sexism for the male leader (indirect effect = 0.58, *SE* = 0.14, 95% CI [0.32, 0.85]), but not for the female leader (indirect effect = 0.10, *SE* = 0.12, 95% CI [-0.15, 0.35]). Specifically, as can be seen in **Figure 1**, participants who saw a male leader display QB-type behavior saw him as having less positive intent (*a* = -1.23), which in turn related to increased attributions to sexism (*b* = -0.47). There was no effect of QB-type behavior on perceived sexism independent of its effect on perceived positive intent (*c*′ = 0.44, *p* = 0.180). Thus, consistent with our expectations, participants perceived a male leader displaying QB-type behavior as more sexist (relative to control) because they perceived him as lacking positive intent.^3^

**Table 3:** Conditional indirect effects of QB(-type) behavior on perceived sexism through perceived positive intent.

	*B*	*SE*	*p*
*Outcome = M (perceived positive intent)*			
Constant	3.84	0.17	<0.0001
QB(-type) behavior (QB)	-1.23	0.24	<0.0001
Gender of leader (GL)	0.13	0.24	0.5795
QB × GL	1.02	0.35	0.0035
	*R*^2^ = 0.215, *F*(3,164) = 14.98, *p* < 0.0001		
*Outcome = Y (perceived sexism)*			
Constant	4.74	0.34	<0.0001
QB(-type) behavior	0.45	0.20	0.0245
Perceived positive intent	-0.47	0.08	<0.0001
	*R*^2^ = 0.250, *F*(2,165) = 27.49, *p* < 0.0001		
*Conditional indirect effects*	Indirect effect	*SE*	95% CI
Gender leader = male	0.58	0.14	[0.32, 0.85]
Gender leader = female	0.10	0.12	[–0.15, 0.35]


The data thus partially supported Hypothesis 1. As expected, a male (but not a female) leader displaying QB(-type) behavior was seen as having less positive intent toward women, and although we did not find a significant difference in the direct effect of QB(-type) behavior on perceived sexism for the male vs. for the female leader, there was a significantly different indirect effect: differences in perceived positive intent indirectly led to differences in perceived sexism.

### How Are Participants Affected by QB(-Type) Behavior?

First, we examined whether exposure to QB(-type) behavior was related to higher negative affect. As expected, there were significant main effects of QB(-type) behavior on anxiety, *F*(1,166) = 5.20, *p* = 0.024, $\eta_{p}^{2}$ = 0.03, anger, *F*(1,166) = 20.92, *p* < 0.001, $\eta_{p}^{2}$ = 0.11, and sadness, *F*(1,166) = 18.19, *p* < 0.001, $\eta_{p}^{2}$ = 0.10. Participants in the QB(-type) conditions reported being more anxious (*M* = 3.80, *SD* = 1.17), more angry (*M* = 3.48, *SD* = 1.36), and more sad (*M* = 3.34, *SD* = 1.03) than did participants in the control conditions (*M* = 3.38, *SD* = 1.18; *M* = 2.57, *SD* = 1.24; *M* = 2.64, *SD* = 1.08, respectively), though power for the main effect on anxiety was rather low (0.62). There were no interactions between leader behavior and leader gender on anxiety, *F*(1,164) = 0.64, *p* = 0.427, $\eta_{p}^{2}$ = 0.004, anger, *F*(1,164) = 0.04, *p* = 0.846, $\eta_{p}^{2}$ = 0.0002, or sadness, *F*(1,164) = 0.02, *p* = 0.887, $\eta_{p}^{2}$ = 0.0001. Thus, supporting Hypothesis 2, QB(-type) behavior related to negative outcomes both when this behavior came from a male leader and when this behavior came from a female leader.

### Is Regulation of Negative Effects Impaired Under a Female Leader?

**FIGURE 2:**
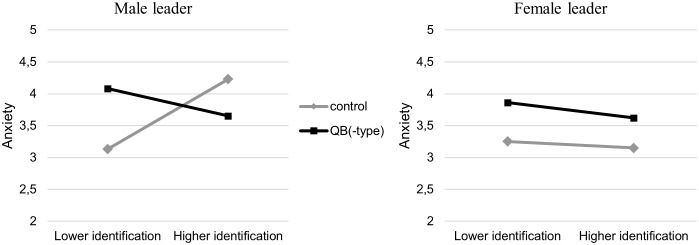
Simple slopes showing anxiety as a function of leader behavior (QB[-type] vs. control) and gender identification (–1 SD, +1 SD) in the male leader and in the female leader condition.

Next, we examined whether gender identification acts as a buffer against the effect of QB(-type) behavior on negative emotions. Using the PROCESS macro for SPSS (Hayes, 2018, model 3), we examined the degree to which gender identification (as a continuous moderator) moderated the effects of leader gender and leader behavior on negative emotions. We expected to find a three-way interaction such that for participants with a male leader, gender identification would serve as a buffer of the effect of QB(-type) behavior on negative emotions, while a similar effect would not occur for participants with a female leader. Results showed that the three-way interaction between leader behavior, leader gender, and gender identification was marginally significant for anxiety, *F*(1,160) = 3.85, *p* = 0.052, $\eta_{p}^{2}$ = 0.02. Further examination of this interaction showed that among participants who had seen a male leader, the main effect of QB-type behavior on anxiety was moderated by gender identification, *b* = -0.75, *F*(1,160) = 9.22, *p* = 0.004. In line with expectations, simple slope analyses looking at participants with lower and higher gender identification (-1 SD and +1 SD) showed that participants lower in gender identification reported more anxiety when their male leader evidenced QB-type behavior than when he evidenced neutral behavior, *b* = 0.82, *p* = 0.016, while participants higher in gender identification reported equal anxiety regardless of whether their male leader evidenced QB-type or control behavior, *b* = -0.43, *p* = 0.189 (see **Figure 2**).^4^ Meanwhile, among participants who had seen a female leader, the effect of QB behavior on anxiety was not moderated by gender identification, *b* = -0.07, *F*(1,160) = 0.08, *p* = 0.773. Contrary to expectations, gender identification and leader gender did not interact with leader behavior to produce significant three-way interactions on anger, *F*(1,160) = 1.82, *p* = 0.179, $\eta_{p}^{2}$ = 0.01, or sadness, *F*(1,160) = 1.40, *p* = 0.239, $\eta_{p}^{2}$ = 0.01. The data thus partially supported Hypothesis 3, cautiously suggesting impaired regulation of negative effects on anxiety (but not anger or sadness) following exposure to QB(-type) behavior from a female source, but not from a male source. However, as this effect was underpowered (0.45), these results should be interpreted with due caution. We further reflect on the issue of power in the discussion section.

## Discussion

While previous research has investigated the occurrence and antecedents of self-group distancing in women (also known as “Queen Bee” behavior; Derks et al., 2011a,b, 2015, 2016), the current study shifted focus from antecedents to subsequent *effects* of this behavior on junior women. Results showed that women perceived a male but not a female leader displaying QB(-type) (vs. neutral) behavior as having less positive intent toward women, which in turn related to stronger attributions of sexism. This finding is consistent with research demonstrating that possible displays of sexism directed toward women are less likely to be noticed when the source of this behavior is a woman (Baron et al., 1991; Barreto and Ellemers, 2005; Cunningham et al., 2009). This is the first research, however, to empirically demonstrate a similarity between QB(-type) behavior and sexism (which, as outlined before, are similar in behaviors but conceptually very different given their different underlying concerns or antecedents). Our results show that, like sexism, QB(-type) behavior negatively affects women (Klonoff et al., 2000; Wang et al., 2012). This study is also the first to examine the impact of possibly biased comments from an ingroup leader to an ingroup subordinate. Moreover, we add to research on effects of possible sexism by male and female sources (Barreto and Ellemers, 2005) by including a male and a female control condition rather than only comparing a male source to a female source. With this condition, we were able to eliminate the alternative explanation that a man displaying QB-type behavior was seen as sexist *only* because of his gender. The results show that a man displaying QB-type behavior was seen as more sexist than a male leader displaying neutral behavior. Thus, beyond a main effect of gender (male leader perceived to be more sexist than a female leader); the act of displaying QB-type behavior uniquely contributed to perceived sexism.

In line with research on the intergroup sensitivity effect (Hornsey et al., 2002; Hornsey and Imani, 2004), participants attributed QB-type behavior coming from a member of the outgroup (a man) to a lack in positive intent toward the ingroup (women), which is why he was seen as sexist. Coming from an ingroup source, however, QB behavior was not attributed to a lack in positive intent. These findings provide further insight into when and why people attribute behavior to bias. Put differently, these findings illustrate circumstances under which people may *not* attribute behavior to bias, that is when behavior is presented in the context of perceived positive intent.

Although an ingroup leader displaying QB(-type) behavior was less likely than an outgroup leader to be viewed in a negative light, participants nonetheless experienced negative consequences of this behavior. In both the male leader and in the female leader conditions, exposure to QB(-type) behavior increased negative emotions. So while participants did not explicitly perceive QB behavior coming from a female leader as having negative intent, participants’ affective responses were as negative as when they had been exposed to a male leader displaying QB-type behavior. Specifically, participants who had been exposed to QB(-type) behavior were more angry, more sad, and more anxious than participants who had not been exposed to this behavior, regardless of leader gender. Notably, as all women in the current study (including those in the control conditions) received ambiguous negative feedback, we can rule out the alternative explanation that it was the act of feeling rejected rather than exposure to QB(-type) behavior which increased negative emotions. As far as we know, this is the first research to show that QB behavior negatively affects junior women’s well-being. The finding that QB behavior does not have to be perceived as negative (i.e., negative intentions or sexist) to exert a negative influence is consistent with research showing that bias does not have to be identified as such to exert negative effects (e.g., Barreto and Ellemers, 2005). These findings suggest that QB behavior affects junior women in a way that may go unnoticed: increasing negative emotions but being less likely to be identified as potentially harmful, thus lowering the opportunity to defend against its effects.

The results indeed suggested that high identifiers–those usually protected from some of the negative consequences of bias, as they are highly vigilant and more confident as to when bias occurs–may not be protected in the usual way against this type of bias. That is: both low and high identifiers showed higher anxiety when exposed to QB behavior by a female leader. Among participants with a male leader, however, higher gender identification buffered against the negative effects of QB-type behavior on anxiety. Yet, this three-way interaction was only marginally significant and underpowered, thus replication research with larger samples is needed to draw more confident conclusions on the regulation of negative effects of QB(-type) behavior by male and female leaders.

Combining the current results with existing research on self-group distancing suggests that self-group distancing behavior in organizations may have a number of negative consequences. These consequences are relevant not only for gender groups, but also for other negatively stereotyped and underrepresented groups. Having an ingroup leader who distances him or herself from the ingroup can have pernicious effects for members of that group. Leaders who show possible bias toward underrepresented groups create a negative work environment for members of these groups, even when they themselves are members of these traditionally underrepresented groups and even when they are not perceived as being biased.

Our results highlight the key importance of the organizational climate in any effort to target underrepresentation of groups in the workplace. Only placing a few more minorities and women in the higher echelons of the organizations is not sufficient without also targeting the organizational diversity climate–or at least not if this increase in women and/or minorities does not lead to a critical mass (Kanter, 1977; Torchia et al., 2011; see also Burkinshaw, 2015). Rather than changing stereotypes or improving diversity, select representation of only a few minorities or women without achieving a critical mass may even increase stereotyping and preferential treatment by the majority group (Wright, 2001; Bagues et al., 2017). Without achieving a critical mass, women or ethnic minorities may continue to adapt to threatening organizational climates by distancing themselves from the stigmatized ingroup, which could have negative effects on future career perspectives for members of these groups. Other than achieving a critical mass, options to break the chain of self-group distancing are to create a more inclusive or otherwise less threatening organizational climate (Purdie-Vaughns et al., 2008) and to ensure that members of stigmatized groups have access to successful role models who they feel similar to and who do not distance themselves from the ingroup (Cheryan et al., 2011).

### Limitations and Future Research

A limitation of the current study is that the three-way interactions between leader gender, leader behavior, and gender identification on negative affect were underpowered. Future research should further investigate whether gender identification is indeed an effective regulation strategy for QB(-type) behavior by a male but not by a female leader. Moreover, since our results suggested that regulation may be different for different negative emotions (marginal effect for anxiety and no effect for anger or sadness), research could examine whether some emotions are more difficult to regulate in reactions to self-group distancing behaviors. Regulation through directing emotions toward others instead of the self is also an interesting route for further research. For instance, it could be that women with higher gender identification regulate anger not by decreasing this emotion, but by directing it toward the leader, while women with lower gender identification may experience anger toward themselves. This interpretation is consistent with research showing that unambiguous and ambiguous bias both increase negative emotions, but that these emotions are more likely to be directed toward the other when bias is unambiguous, and toward the self when bias is ambiguous (Crocker et al., 1991; Vorauer and Kumhyr, 2001; Ellemers and Barreto, 2009; Barreto et al., 2010).

The present study manipulated QB(-type) behavior with its three main components shown in previous research (masculine self-description, endorsing gender stereotypes and denying gender discrimination). Future research could investigate whether some of these components are more influential than others. It would also be interesting to study whether self-group distancing not only affects subordinates’ negative emotions, but perhaps also harms organizational outcomes such as employee satisfaction, organizational commitment, or productivity, especially for members of the negatively stereotyped group. Additionally, future research can examine long-term consequences of leader self-group distancing for subordinates of negatively stereotyped groups. These consequences may include subordinates switching to other careers where they might feel more belonging (Drury et al., 2011; Veldman et al., 2017) or adjusting to the organizational climate by engaging in self-group distancing themselves.

Another avenue for future research could be to examine the processes through which QB(-type) behavior induces negative affect, as it is possible that these processes are different for male and for female leaders, or that for female leaders additional processes are at play. For instance, junior women exposed to QB-type behavior by a male leader may experience more negative affect because they suspect they are a victim of discrimination. Junior women exposed to QB behavior by a female leader may experience negative affect through other or additional processes, for instance because they do not see this female leader as a role model and may fear that success is attainable only for women who are dissimilar to them. Indeed, research has shown that for members of underrepresented groups, a role model who embodies qualities stereotypical of a particular field (i.e., masculine in male-dominated field) may even be less desirable than not having a role model at all (Cheryan et al., 2011). Insight into these processes would strengthen the present research by revealing the underlying mechanisms behind negative effects of QB behavior, and may provide ways to protect junior women from such effects.

It would be interesting to examine self-group distancing and its consequent negative effects in different groups. For instance, would similar effects be found among men employed in traditionally female-dominated work environments? Men in these fields may be underrepresented but not necessarily negatively stereotyped, however, and any negative gender stereotyping there might be is likely to affect men less (Schmitt et al., 2002). As such, men may suffer less from identity threat in these contexts and may be less likely to have to resort to a strategy such as self-group distancing. Moreover, men who *do* distance themselves from other men may suffer a loss of status in the eyes of other men and may therefore be less influential (thus exerting less negative effects). The same results might thus not be found among men. We would certainly though expect similar results to be found among other negatively stereotyped groups, such as ethnic minority groups. Here, too, we expect that self-group distancing from an ingroup source may not be identified as bias and may have similar negative consequences, including impaired regulation of these consequences.

## Conclusion

Existing work shows that an organizational climate that is not identity safe can trigger self-group distancing in members of negatively stereotyped groups. The current work adds that behavior associated with self-group distancing might not be recognized as bias when coming from a member of the ingroup, but nevertheless negatively affects members of that group, making it potentially more likely that members of negatively stereotyped groups will feel lower belonging and motivation. To put it a different way, gradual advancement of members of underrepresented groups will not necessarily lead to increased equality for these groups as long as the environment these individuals advance in leads them to distance themselves from their group. Importantly, these findings do not mean that women and other members of disadvantaged groups should not advance into higher organizational positions. Rather, it is key that efforts also be directed toward removing the structural barriers and the lack of positive climate that members of disadvantaged groups in these positions can face, thus alleviating the need for members of negatively stereotyped and underrepresented to cope by engaging in self-group distancing.

## Ethics Statement

This study was carried out in accordance with the recommendations of the General Guidelines, Social and Societal Ethics Committee of the University of Leuven. The protocol was approved by the Social and Societal Ethics Committee of the University of Leuven. All subjects gave written informed consent in accordance with the Declaration of Helsinki. Before starting the questionnaire, participants agreed to an informed consent that provided the general research aims. They were informed that participation was voluntary and could be stopped at any moment during the study; and that their responses are anonymous and treated confidentially. Moreover, they were provided with room for questions and comments as well as all contact information of the researchers and the ethical committee.

## Author Contributions

NS, CVL, and LM contributed to the development of hypotheses, data collection, interpretation of results, and the writing of the manuscript. NS conducted the statistical analyses.

## Conflict of Interest Statement

The authors declare that the research was conducted in the absence of any commercial or financial relationships that could be construed as a potential conflict of interest.

## Footnotes

1 The interpretation of the results is by and large the same with and without exclusions: without exclusions, the interaction effect between leader gender and leader behavior on perceived sexism approaches significance more strongly (*p* = 0.072 without exclusions versus *p* = 0.126 with exclusions) and has more power (0.436 without exclusions versus 0.333 with exclusions). All results pertaining to the rest of H1 (mediation through intent), H2 (negative effects) and H3 (regulation of effects) remain unchanged.

2 One additional measure, attributional ambiguity, examined the extent to which participants were (un)sure about their judgment of sexism of their leader. The results did not substantially add to the story while decreasing the coherence thereof, which is why these results are not included in the main text. The measure as well as the results of analyses with this measure are provided in the **Supplementary Materials**. At the end of the study, other measures were administered for exploratory purposes for future research (participants’ masculine and feminine self-description, interest in individual mobility, and distancing from the group). These measures were unrelated to the research questions or hypotheses described in this manuscript, and were administered after the measures relevant to the present study had been administered. Thus, these measures did not exert any influence on the present results and can be seen as separate from the present results.

3 We also tested the alternative reversed mediation, where QB-type behavior through the mediator of perceived sexism would lead to decreased positive intent. This moderated mediation model was not significant, index = -0.22, *SE* = 0.16, 95% CI [-0.06, 0.56]. Moreover, the–albeit significant–indirect effect of QB-type behavior on perceived positive intent through the mediator perceived sexism in the male leader condition (indirect effect = -0.38, *SE* = 0.13, 95% CI [-0.66, -0.15]) did not fully mediate the effect of QB-type behavior on perceived sexism (*c*′ = -0.96, *p* < 0.001).

4 See **Supplementary Materials** for figures displaying the spread of datapoints around the slopes (**Supplementary Figure A**).
