# Supplementary figures and images for: Perpetuating Inequality: Junior Women Do Not See Queen Bee Behavior as Negative but Are Nonetheless Negatively Affected by It
Source: Front Psychol. 2018 Sep 20;9:1690. doi: 10.3389/fpsyg.2018.01690 (PMC6159757; doi:10.3389/fpsyg.2018.01690)

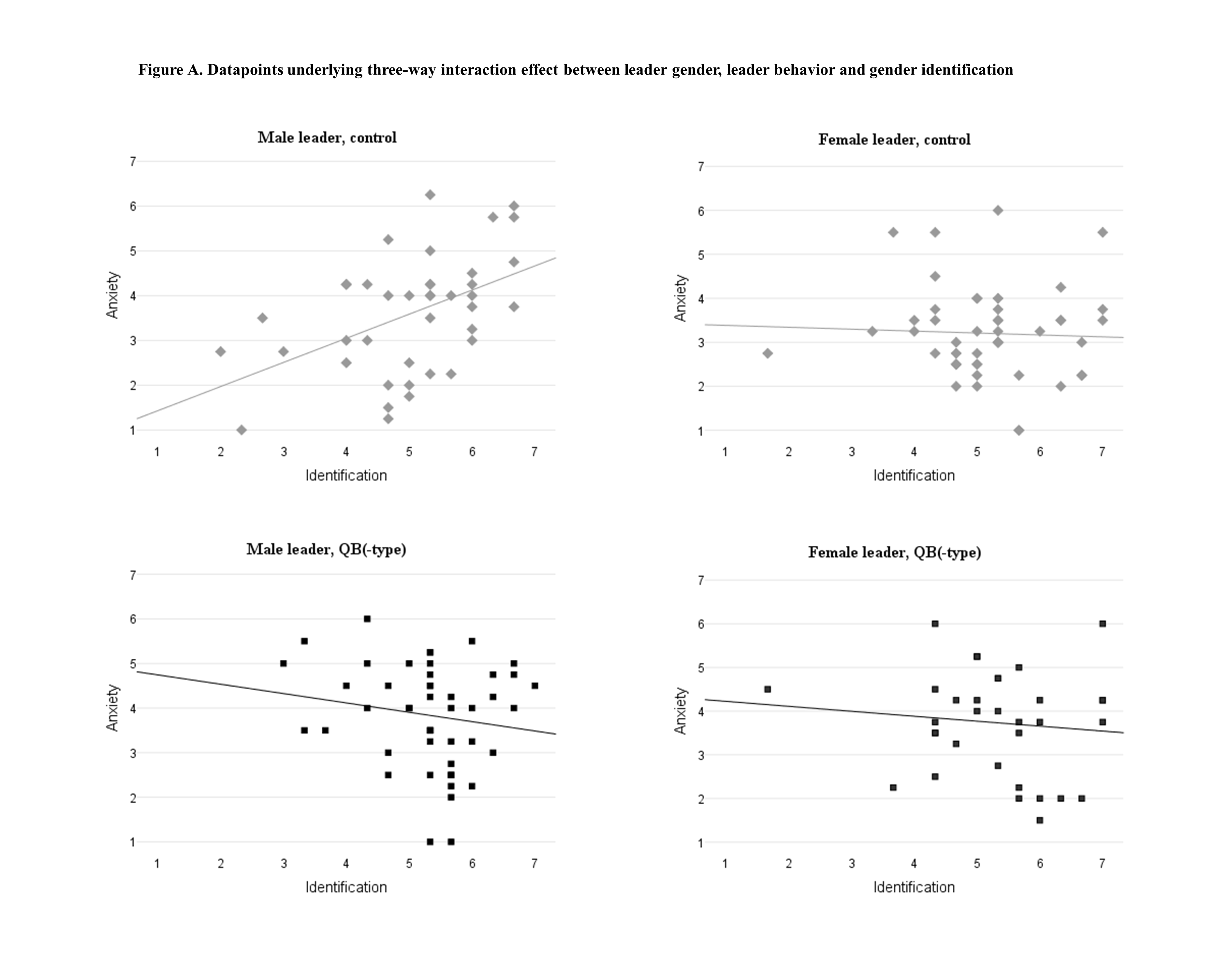

Supplement: Supplementary file 1 [file Image_1.TIF]
