# Supplementary material for: Perpetuating Inequality: Junior Women Do Not See Queen Bee Behavior as Negative but Are Nonetheless Negatively Affected by It
Source: Front Psychol. 2018 Sep 20;9:1690. doi: 10.3389/fpsyg.2018.01690 (PMC6159757; doi:10.3389/fpsyg.2018.01690)
Supplement: Supplementary file 2 [file Table_1.docx]

Supplementary Material

Perpetuating Inequality: Junior Women Do Not See Queen Bee Behavior as Negative but Are Nonetheless Negatively Affected by It

Naomi Sterk, Loes Meeussen, and Colette Van Laar

*** Correspondence:** Naomi Sterk: [naomi.sterk@kuleuven.be](mailto:naomi.sterk@kuleuven.be)

**Table of contents**

[1 Manipulation 2](#_Toc523610672)

[1.1 Introduction CEO (male-dominated organizational context) 2](#_Toc523610673)

[1.2 QB-type behavior manipulation text (female leader condition) 3](#_Toc523610674)

[1.3 Control condition manipulation text (male leader condition) 4](#_Toc523610675)

[2 Attributional ambiguity 4](#_Toc523610676)

[3 Statistics for ART analyses 6](#_Toc523610677)

# Manipulation

## Introduction CEO (male-dominated organizational context)


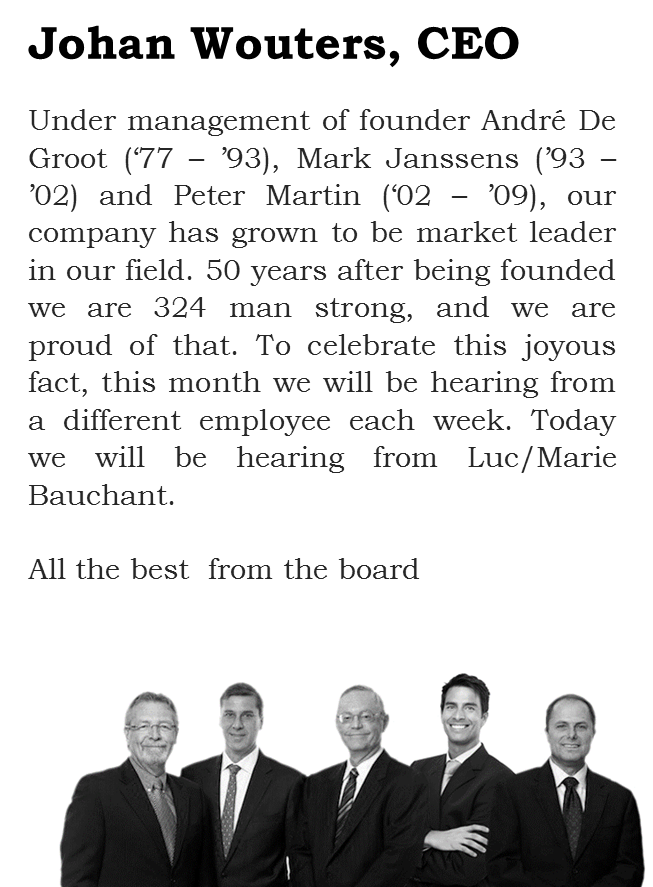


## QB-type behavior manipulation text (female leader condition)


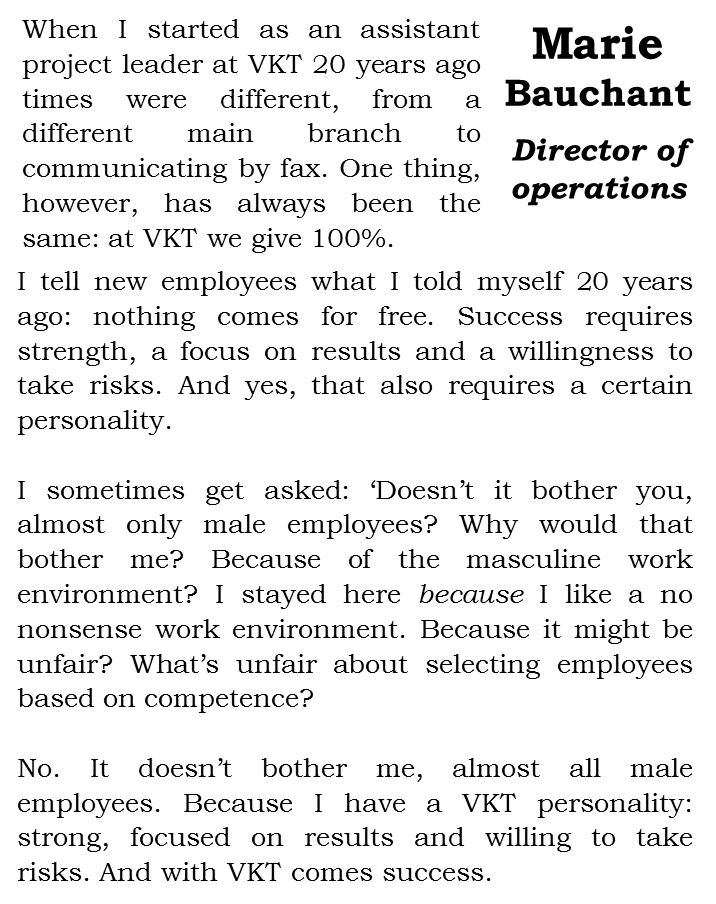


## Control condition manipulation text (male leader condition)


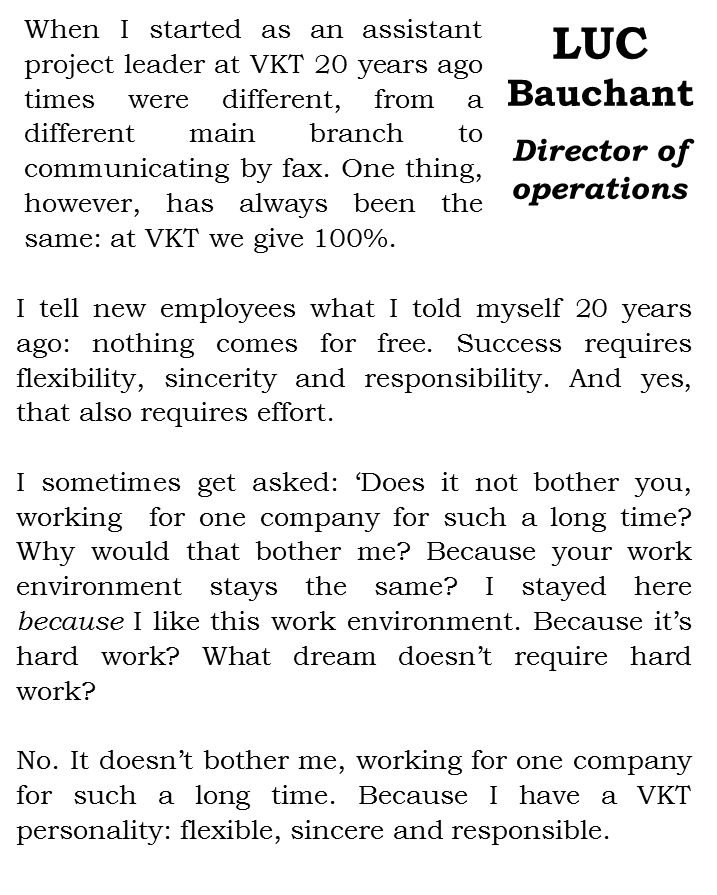


# Attributional ambiguity

*Attributional ambiguity*. To measure attributional ambiguity, participants were presented with the preceding question about their impression of sexism of the leader (“Luc/Marie is sexist”), as well as with their response to that statement. They were asked to indicate how sure they were of their rating of perceived sexism. Participants answered on a 7-point Likert scale from (1) very unsure to (7) very sure. Responses were recoded so that a higher score reflects more ambiguity (more unsure).

We tested whether participants in the female QB condition would report more ambiguity regarding the extent to which they found their leader to be sexist (relative to the control condition and the male QB-type behavior condition). The results showed a significant two-way interaction between leader behavior and leader gender on ambiguity, *F*(1, 164) = 10.98, *p* = .001, *η_p_²* = .06. The simple main effect of leader behavior on ambiguity was not significant for participants exposed to a female leader, *F*(1, 164) = 1.39, *p* = .241. Among participants who had seen a male leader, the simple main effect of leader behavior on ambiguity was significant, *F*(1, 164) = 36.55, *p* < .0001, *η_p_² =* .18, such that participants were less sure (more ambiguity) of their sexism ratings for the male leader displaying neutral behavior than for the male leader displaying QB-type behavior. Given that participants rated the male leader as more sexist than the female leader regardless of behavior (main effect of leader gender on sexist attributions, see manuscript), this additional effect on attributional ambiguity indicates that participants were more sure of this attribution of sexism when they had seen a male leader displaying QB-type behavior than when they had seen a male leader displaying neutral behavior.

Examined the other way, the significant interaction-effect by leader behavior showed that in the control conditions (neutral leader behavior), ambiguity was higher under a male leader (*M* = 5.23, *SD* = 1.72) than under a female leader (*M* = 4.42, *SD* = 2.07, *p* = .037, *η_p_² =* .03). In the QB-type behavior conditions, the direction of this effect (the simple main effect of leader gender) was reversed and ambiguity was higher under a female leader (*M* = 3.94, *SD* = 1.66) than under a male leader (*M* = 2.89, *SD* = 1.63, *p* = .011, *η_p_² =* .04). Thus, when leaders displayed neutral behavior, participants were less sure about attributions of sexism for the male leader than for the female leader (and ratings of sexism were higher for the male than for the female leader, see manuscript). Conversely, participants were less sure of their attributions of sexism when they had seen a female leader displaying QB behavior than when they had seen a male leader displaying QB-type behavior.

# Statistics for ART analyses

Main effects of leader gender and leader behavior on perceived sexism

| Source | Type III SS | df | *F* | *p* | η^2^ |
| --- | --- | --- | --- | --- | --- |
| Leader behavior | 23722.58 | 1 | 12.04 | .0007 | .068 |
| Leader gender | 35502.72 | 1 | 18.02 | < .0001 | .098 |
| Error | 325062.27 | 165 |  |  |  |
| Total | 1592766 | 168 |  |  |  |
|  |  |  |  |  |  |

Interaction effect of leader gender and leader behavior on perceived sexism

| Source | Type III SS | df | *F* | *p* | η^2^ |
| --- | --- | --- | --- | --- | --- |
| Interaction | 5053.46 | 1 | 2.13 | .146 | .013 |
| Error | 389137.71 | 164 |  |  |  |
| Total | 159394 | 168 |  |  |  |
|  |  |  |  |  |  |
